# Cancer survivors’ perspectives on delivery of survivorship care by primary care physicians: an internet-based survey

**DOI:** 10.1186/s12875-015-0367-x

**Published:** 2015-10-20

**Authors:** Ernestina Nyarko, James M. Metz, Giang T. Nguyen, Margaret K. Hampshire, Linda A. Jacobs, Jun J. Mao

**Affiliations:** Perelman School of Medicine at the University of Pennsylvania, Pennsylvania, USA; Department of Radiation Oncology, University of Pennsylvania, Pennsylvania, USA; Department of Family Medicine and Community Health, University of Pennsylvania, 227 Blockley Hall, 423 Guardian Drive, Philadelphia, PA 19104-6021 USA; Abramson Cancer Center, University of Pennsylvania, Pennsylvania, USA; Center for Clinical Epidemiology and Biostatistics, University of Pennsylvania, Pennsylvania, USA

**Keywords:** Cancer, Survivor, Perspective, Primary Care, Internet, Survey, Race, Quality of Care, Trust

## Abstract

**Background:**

Helping cancer survivors to transition from active treatment to long-term survivorship requires coordinated efforts by both oncologists and primary care physicians (PCPs). This study aims to evaluate cancer survivors’ perspectives on PCP-delivered survivorship care.

**Methods:**

We conducted an Internet-based cross-sectional survey of cancer survivors via www.OncoLink.org. Regression analyses were used to identify factors associated with perception of PCP-delivered survivorship care.

**Results:**

The 352 respondents rated overall PCP-delivered survivorship care as 60 out of 100 (SD = 23). The areas of care most strongly endorsed were general care (62 %), psychosocial support (65 %), and holistic care (68 %). Survivors were less likely to perceive their PCPs as knowledgeable about cancer follow-up (43 %), late or long-term effects of cancer therapy (45 %), and diagnosis and treatment of symptoms related to cancer or cancer therapy (42 %). While 72 % of survivors reported satisfaction with their PCP’s care overall, only 41 % felt that their PCPs and oncologists communicated well with one another. In a multivariate regression analysis, higher trust in PCP (*p* < 0.001), non-white race (*p* = 0.001), living in the United States (*p* = 0.007), and visiting a PCP two or more times per year (*p* = 0.009) were significantly associated with higher ratings of PCP-delivered survivorship care.

**Conclusions:**

While cancer survivors in general are satisfied with care delivery by PCPs, they perceived that their PCPs have limited abilities in performing cancer-specific follow-up and late effect monitoring and treatment. Better education of family physicians about survivorship issues and improved communication between PCPs and oncologists are needed to improve PCPs’ delivery of survivorship care.

## Background

With improved surveillance, early detection, and treatment of cancer, the number of cancer survivors living beyond the active treatment phase is increasing in the United States. As of 2007, there were 12 million cancer survivors living in the United States, up from 9.8 million in 2001 and 3 million in 1971. The largest groups of cancer survivors were female breast cancer survivors (22.1 %), prostate cancer survivors (22.1 %), and colorectal cancer survivors (9.5 %) [1]. As cancer survivors live decades beyond their initial cancer diagnosis, they are at risk for developing a number of physical and psychosocial conditions and late or long-term effects that are independent of their cancer or treatment. Survivors may experience chronic pain, fatigue, sleep problems, cognitive issues, sexual dysfunction, ongoing depression/distress, fear of recurrence, and changes in their overall quality of life [2]. Studies in breast cancer survivors have found high prevalence of these symptoms among survivors [3–6]. Lung, colon, and prostate cancer survivors have also reported significant and persistent physical, psychosocial, sexual, and marital problems for years after disease remission that significantly impact their quality of life [7].

Traditionally, cancer patients receive healthcare through medical, surgical, or radiation oncologists for issues that arise during active cancer treatment and the initial post-treatment surveillance period. Thus, these providers have been thought of as the first line in addressing survivors’ needs when cancer-related or treatment-related sequelae arise. However, studies show that while cancer survivors visit oncologists frequently during active treatment, the percentage of survivors who visit oncologists and cancer-related specialists declines each year following active treatment. While survivors visit their oncologists less and less as time passes, the percentage of survivors who visit their primary care physician (PCP) each year is consistently high at around 75 % and does not decline as survivors get further out from initial diagnosis [8, 9]. This means that as time progresses, the vast majority of cancer survivors will present to their PCPs instead of their oncologists for evaluation and management of late and long-term effects. The PCP can play a critical role in the management of a cancer survivor’s health by delivering preventive and general care as well as managing co-morbid conditions that may be unrelated to the cancer. Consequently, a team effort is required in order to assure that relevant information regarding cancer treatment, surveillance schedule, and general survivorship issues is communicated to the PCP by the oncology team [10].

Unfortunately, the transition of care from oncology to primary care is often fragmented and uncoordinated. A 2005 Institute of Medicine (IOM) report revealed that many cancer survivors felt uncertain about the care they receive once they transition from the care of their oncologists at the end of their cancer treatment course. Consequently, the IOM recommended a cancer treatment survivorship care plan for cancer survivors during the transition period that outlines information for long-term care including cancer diagnosis, treatment, potential consequences of treatment and guidelines for follow-up visits, general tips on healthy living and prevention of new and recurrent cancers, and the availability of psychosocial and legal support services [11]. Interestingly, research done to date demonstrates that family physicians are able to provide care that is similar in quality and outcomes as that provided by oncology specialists for breast cancer survivors [12]. However, a recent study found that survivorship care planning did not improve patient-reported outcomes and health care service utilization compared to standard care in the same type of population [13, 14]. In order to deliver the comprehensive, coordinated care suggested by the IOM, PCPs must become more comfortable with the delivery of cancer survivorship care. Since 2005, several surveys of primary care physicians and oncologists have been conducted which indicate that both PCPs and oncologists acknowledge the importance of primary care physician involvement in new models of survivorship care delivery and are willing to work toward improving the transition of care [15–21].

While it is important to understand the views of PCPs and oncologists regarding the transfer of cancer survivorship care from primarily oncology-based to PCP-based, it is vital to incorporate cancer survivor perspectives as we move toward designing and implementing new models of patient-centered survivorship care. To date, few studies have examined cancer survivor perspectives on the delivery of survivorship care by primary care physicians. The few studies of survivor perspectives of PCP-delivered survivorship care have focused primarily on patients presenting to urban or university-affiliated cancer centers [22–24]. In this study, we used an Internet-based survey to quantify cancer survivor perspectives of PCP-delivered survivorship care. Our aim in using an Internet-based survey was to gather information from more geographically diverse groups of cancer survivors underrepresented in the current literature.

## Methods

### Study design and respondent population

Between July 2009 and June 2012, an Internet-based questionnaire assessing patient demographics and perceptions of primary care physician-delivered survivorship care was intermittently (few days every few months) posted on the OncoLink website (http://www.oncolink.org). OncoLink, one of the oldest and largest Internet-based cancer information resources, serves close to 1.5 million web pages and receives more than 189,000 visits from unique IP addresses per month. OncoLink is available to anyone who has access to the Internet. Currently, 75 % of OncoLink’s user base is from the United States, while 25 % access the website from international locations [25].

The study questionnaire was composed of 30 items assessing patient demographics, primary cancer diagnoses, treatment modalities used, setting of cancer treatment, use of primary care and specialty care, relationship with primary care physician and perceptions of survivorship care delivery by primary care physicians. The questionnaire was randomly rotated on the homepage and other high profile locations on the website. There was no use of external advertising or directed e-mail announcements. Study participants voluntarily and anonymously completed the survey instrument after reading an online informed consent document. Survey questions and responses were maintained on a physically and electronically secure server and downloaded for analysis. This cross-sectional study was approved by the University of Pennsylvania Institutional Review Board.

### Primary outcome measure

The primary outcome measure was a seven-item Primary Care Delivery of Survivorship Care Scale (PCDSCS) [24]. Each item on the PCDSCS is scored on a 5-point Likert scale from 0 to 4. The scale scores had an excellent internal consistency with a Cronbach’s alpha of 0.89. Principal component analysis yielded one factor with eigenvalue of 3.89. The higher score was correlated with greater trust in PCPs. The total score was transformed and placed on a 100-point scale with higher number indicating better perceived care delivery by PCPs [24].

### Covariates

Respondents reported demographic, cancer-related, and healthcare-related variables. Demographic variables included age, sex, race, educational level, residential area, and country of residence. Cancer-related variables included type of cancer, type of treatment (s) received, and age at cancer diagnosis. Healthcare-related variables included type of physician managing care, whether the respondent has been offered survivorship care, distance to and setting of the respondent’s cancer treatment center, and length and frequency of relationship with their primary care physician. Respondents also rated communication between their PCP and oncologist and trust that their PCP works toward the respondent’s best interests as a patient. Trust in PCP was rated on a numerical scale from 1 to 10 with 10 indicating highest trust. Because this variable was not normally distributed, we dichotomized at the median value as high vs. low trust.

### Statistical analysis

Statistical analysis was performed using STATA 12.0 for Windows (STATA Corp, College Station, TX). Bivariate regression analysis was used to select patient characteristics associated with the outcome variable, PCDSCS. Variables that showed a strong association (*p* < 0.1) were then used to generate a multivariate regression model to further characterize patient variables associated with perceived survivorship care delivery by PCPs. All analyses were two-sided with *p* < 0.05 indicating statistical significance. Chi-square analyses of individual PCDSCS statement endorsement were performed to determine which aspects of PCP survivorship care delivery might explain significant differences observed between groups in multivariate regression. Respondents were considered to endorse a particular aspect of PCP survivorship care delivery if they answered “Agree” or “Strongly agree” in response to the corresponding item on the survey instrument.

## Results

### Respondent characteristics

The mean age for the 352 cancer survivors who responded to the Internet survey was 54.1 years. Two hundred and ninety-three respondents (83.5 %) were female and 58 (16.5 %) were male. Racially, 277 (79.8 %) were Caucasian, 36 (10.4 %) were Asian or Pacific Islander, 15 (4.3 %) were African-American, 9 (2.6 %) were Hispanic or Latino, 6 (1.7 %) were of mixed race, and 4 (1.2 %) considered themselves to be of another race. One hundred and thirty-six (39.3 %) lived in urban areas, 151 (43.6 %) lived in suburban areas, and 59 (17.1 %) lived in rural areas. Two hundred and thirty-two (66.5 %) respondents held a college degree (BA, BS) or higher. Respondents included survivors of thirty different cancer types. Of these, 149 (43.1 %) were breast cancer survivors, 19 (5.5 %) were lung cancer survivors, 18 (5.2 %) were colon cancer survivors, 15 (4.3 %) reported a history of ovarian cancer, and 11 (3.2 %) were prostate cancer survivors. One hundred and thirty-seven (39.9 %) received cancer care exclusively at university-based cancer centers, while 150 (43.7 %) reported receiving cancer care at community-based non-university affiliated centers. Two hundred and eighty-two respondents (81.5 %) were living in the United States, 8 (2.3 %) were living in Canada, and 56 (16.2 %) reported living in sixteen other countries. Respondents living in the United States were from forty of the fifty states and represented all four census regions of the country. Of the 243 respondents who indicated their state of residence, 88 (36.2 %) were from the Northeast, 63 (25.9 %) were from the West, 57 (23.5 %) were from the South, and 35 (14.4 %) were from the Midwest. For data analysis purposes, race was dichotomized as Caucasian or non-Caucasian and cancer type was dichotomized as breast or non-breast to enhance the power when analyzing these variables.

### Respondent rating of Primary Care Delivery of Survivorship Care Scale (PCDSCS)

Overall, respondents rated primary care physician delivery of survivorship care 59.8 out of 100 (median 60.7, SD 22.7). Respondents most strongly endorsed primary care physician delivery of holistic care (66.8 %), psychosocial support (64.2 %), and general care (61.1 %) that enables patients to live healthier lives. Respondents were less likely to perceive PCPs as knowledgeable in the following domains of care: appropriate cancer follow-up care (42.3 %), late or long-term effects of cancer therapy (44.3 %), and diagnosis and treatment of symptoms related to cancer or cancer therapy (41.8 %). Only 40.3 % of respondents believed that their PCP communicates well with their oncologists in managing their care. Respondent trust in their PCP was high with a mean of 7.54 and a median of 8 (SD 2.64). Trust scores ranged from 0 to 10, with 30.11 % of respondents rating trust in their PCP as 10 out of 10.

### Respondent characteristics associated with PCDSCS score

Table 1 shows the distribution of respondents and PCDSCS scores. In bivariate analysis, non-white race (*p* = 0.004), US residence (*p* = 0.001), and history of radiation therapy (*p* = 0.018) were significantly associated with higher PCDSCS scores. More frequent PCP visits (*p* = 0.002), higher trust in PCP (*p* < 0.001), and perceiving the PCP as one of the people with primary responsibility for patient care (*p* < 0.001) were also associated with higher PCDSCS scores. Interestingly, the setting of cancer treatment and type of cancer (breast or non-breast) were not significantly associated with PCDSCS rating.

**Table 1:** PCDSCS Score and Respondent Characteristics

Characteristic	Patients		Mean PCDSCS score	*P*-value
	No.	%		
Age				0.34
18–39	69	21.2	57.8	
40–64	203	62.5	58.6	
65 or older	53	16.3	61.9	
Gender				0.92
Male	58	16.5	59.6	
Female	293	83.5	60.0	
Race				0.0035
White	277	79.8	58.1	
Non-White	70	20.2	67.0	
Education level				0.32
Less than college	117	33.5	58.2	
College degree (BA/BS) or higher	232	66.5	60.8	
Residence				0.062
Urban	136	39.3	63.1	
Suburban	151	43.6	56.8	
Rural	59	17.1	59.1	
Country				0.0007
United States	282	81.5	61.8	
Outside United States	64	18.5	51.1	
Cancer type				0.75
Breast	149	43.1	60.3	
Non-breast	197	56.9	59.5	
Cancer center				0.65
University-based	137	39.9	58.6	
Community-based	150	43.7	61.1	
Both	56	16.3	59.7	
Chemotherapy				0.55
Yes	196	57.0	60.8	
No	148	43.0	59.3	
Radiation therapy				0.018
Yes	168	48.8	57.1	
No	176	51.2	63.0	
Surgery				0.088
Yes	212	61.6	58.5	
No	132	38.4	62.8	
Hormone therapy				0.36
Yes	106	30.8	58.4	
No	238	69.2	60.9	
PCP visits per year				0.0015
Fewer than 2	101	28.9	53.6	
2 or more	248	71.1	62.2	
Trust in PCP				<0.001
Low	138	39.2	43.5	
Higher	214	60.8	70.3	
Care providers				0.059
PCP/Internist	68	21.3	63.6	
Oncologist	124	38.9	57.5	
Combination	127	39.8	63.6	
Years known PCP				0.008
Less than 1 year	63	18.5	52.5	
1–4 years	108	31.8	64.6	
5–9 years	76	22.4	60.3	
10 or more years	93	27.4	58.5	

Abbreviations: *PCDSCS* primary care delivery of survivorship care scale, *PCP* primary care physician, *BA/BS* bachelor of arts or bachelor of science

**Fig. 1:**
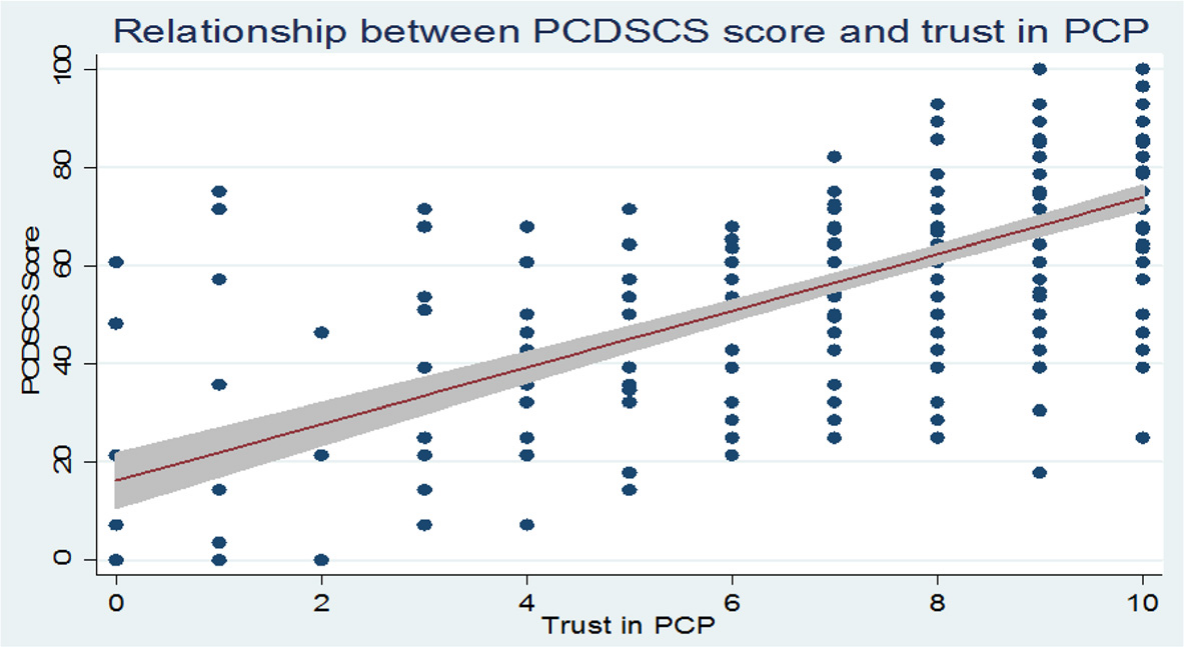
Relationship between PCDSCS score and trust in PCP

In multivariate analysis, non-white race, US residence, and having two or more visits with the PCP annually were significantly associated with higher PCDSCS score (*p* = 0.001, 0.007 and 0.009 respectively). Higher trust in PCP was strongly associated with higher PCDSCS score (*p* < 0.001, Fig. 1). Table 2 depicts the results of multivariate regression analysis.

**Table 2:** Multivariate Regression Analysis of Respondent Characteristics and PCDSCS Score

	Unadjusted analysis			Adjusted analysis		
Characteristic	Coefficient	95 % CI	*P*-value	Coefficient	95 % CI	*P*-value
Race						
Caucasian (reference)						
Non-Caucasian	8.93	2.95 to 14.91	0.004	9.74	3.9 to 15.6	0.001
Residence						
Urban (reference)						
Suburban	−6.32	−11.6 to −1.0	0.019	−2.00	−6.5 to 2.5	0.39
Rural	−3.96	−10.9 to 3.0	0.26	−1.65	−7.8 to 4.5	0.60
Country						
United States (reference)						
Outside United States	−10.71	−16.8 to −4.6	0.001	−7.94	−13.6 to −2.2	0.007
Radiation therapy						
Yes	−5.86	−10.7 to −1.0	0.018	−3.88	−8.0 to 0.3	0.069
No (reference)						
Surgery						
Yes	−4.34	−9.3 to 0.6	0.088	−1.88	−6.4 to 2.6	0.41
No (reference)						
PCP visits per year						
Fewer than 2 (reference)						
2 or more	8.57	3.3 to 13.8	0.002	6.22	1.6 to 10.9	0.009
Trust in PCP						
Low (reference)						
High	26.81	22.8 to 30.8	<0.001	26.47	22.1 to 30.8	<0.001
Care provider						
PCP only (reference)						
Oncologist only	−6.14	−12.8 to 0.5	0.07	−4.91	−10.2 to 0.4	0.070
Both PCP and Oncologist	−0.01	−6.6 to 6.6	1.00	−3.04	−8.4 to 2.3	0.26
Years known PCP						
Less than 1 year (reference)						
1-4 years	12.16	5.1 to 19.2	0.001	2.78	−3.2 to 8.7	0.36
5-9 years	7.85	0.4 to 15.3	0.04	−0.70	−7.2 to 5.8	0.83
10 or more years	6.04	−1.1 to 13.2	0.099	−5.04	−11.6 to 1.5	0.13

Abbreviations: *PCDSCS* primary care delivery of survivorship care scale, *PCP* primary care physician

### *Respondent Endorsement of Individual Aspects of PCDSCS (*Table 3)

**Table 3:** Respondent Endorsement of Individual Aspects of Primary Care Delivery of Survivorship Care

	Race (%)			Country (%)			Trust (%)		
*My primary care doctor…*	White	Non-White	*P*-value	US	Non-US	P-value	High	Low	*P*-value*
Communicates well with my oncologist since my cancer diagnosis	38.3	54.4	0.016	41.9	34.5	0.27	56.1	17.8	<0.001
Is knowledgeable about appropriate follow-up for cancer survivors	38.9	60.3	<0.001	44.6	37.5	0.30	60.6	15.6	<0.001
Is aware of potential long-term effects of cancer treatment	42.3	61.8	0.004	47.6	37.5	0.14	59.9	24.4	<0.001
Is skilled at diagnosing and treating symptoms associated with cancer or cancer therapy	38.5	61.8	0.001	43.5	42.2	0.85	54.9	24.4	<0.001
Pays attention to my emotional wellbeing	65.1	70.6	0.39	68.7	54.7	0.033	84.0	37.8	<0.001
Helps me live a healthier life by discussing diet, exercise, and weight management	60.6	72.1	0.080	68.2	39.1	<0.001	77.8	38.5	<0.001
Is sensitive to my needs as a whole person	69.0	75.0	0.33	72.6	57.8	0.021	87.3	43.0	<0.001

Abbreviation: *US* United States

*P-values calculated by Chi-square analysis

Chi-square analysis showed significant differences between white respondents and non-white respondents in perceived cancer-specific care; scores on general care and communication with oncologists did not differ significantly by race. United States residents were significantly more likely to agree that their PCP helps them live a healthier life by discussing diet and exercise weight management, but did not differ significantly from non-US residents in the endorsement of other statements on PCP delivery of survivorship care. High trust in PCP showed the strongest relationship with individual statement endorsement; respondents reporting higher trust in their PCP rated all aspects of PCP care delivery significantly higher than respondents reporting lower trust in their PCP.

## Discussion

Although there is much interest in the literature on constructing new models of cancer survivorship care, few studies have quantified cancer patients’ perspectives of survivorship care by primary care physicians who are the principal caretakers of cancer survivors after the initial treatment and surveillance period. We found that survivors generally believe that their PCPs provide high quality primary care, psychosocial support, and holistic care, which is consistent with a prior trial demonstrating that breast cancer survivors had high satisfaction with their PCPs [26] and experienced a similar health-related quality of life as if they were cared by specialists [12]. However survivors were less confident in their PCPs’ ability to provide knowledgeable, appropriate cancer-specific care. These findings are consistent with previous studies of breast and prostate cancer survivors[24, 27], emphasizing the need for improving education for PCPs on cancer-specific follow up care.

This study found that non-white patients and patients who visit their PCP more frequently rate PCP delivery of survivorship care significantly higher. The association between non-white race and PCDSCS score may be explained by differences among white patients and minority patients in what they consider to be important components of quality healthcare. A recent study shows that while patients of all racial groups perceive short waiting times, good patient-provider rapport, physician competence, and respect for the patient as important components of quality healthcare, minority patients also cited holistic care that addresses their physical, spiritual, and emotional needs as an important component of quality healthcare [28]. While, the provision of holistic or whole-person care as opposed to disease or organ-specific care may be more appreciated by non-white patients and may contribute to higher ratings of the PCDSCS score, our results also show that non-white patients rate PCP delivery of cancer-specific care more highly than white patients, a finding that calls for further exploration.

The relationship between PCDSCS score and the frequency of visits to the primary care physician can be explained in several ways. More frequent visits with the PCP allow more opportunities for building trust and rapport between patients and their PCP, which likely explains some of our observed association. Furthermore, the literature shows that more frequent PCP visits may be associated with receipt of more appropriate care, which would increase patient satisfaction. For example, a study of prostate cancer patients showed that patients who visited their PCP more frequently were more likely to receive influenza vaccinations, colorectal cancer screening, and cholesterol screening at recommended intervals [29].

An interesting new finding of this study is the significantly higher rating of PCDSCS by survivors living in the United States when compared with those living outside the US. While there may be differences in perceptions of what constitutes high quality care in different countries [30], this finding merits further study if we hope to design successful models of patient-centered survivorship care that will be applicable worldwide. Given that each country has unique health care delivery and health service reimbursement systems, it is likely one size won’t fit all. However, the importance of trust between a physician and patient transcends cultures and health systems. Our study found that survivors generally trust their primary care physicians. If educational systems or institutions provide pathways for PCPs to increase their knowledge of cancer-specific follow-up, surveillance, and diagnosis and treatment of late effects, the high level of trust survivors have in their PCPs will do much to advance survivor acceptance and increase survivor perceptions of PCP-delivered survivorship care.

Overall, this study indicates that cancer survivors have unfavorable perceptions of cancer-specific survivorship care delivered by PCPs. This finding is consistent with previous studies [19, 24, 27, 31] which show that although PCPs are willing to assume more responsibility for cancer survivorship care, they themselves feel that their training and familiarity with cancer surveillance guidelines are inadequate [16, 19, 20, 31–33]. Intervention studies have shown that survivorship care algorithms [12] and survivorship care courses [34] significantly improve PCP comfort with, and delivery of, appropriate cancer survivorship care. Future research should focus on developing models for training both upcoming and practicing PCPs in survivorship care delivery that can be implemented in various practice settings. Further studies should help elucidate the effect of this training on patient perceptions of PCP survivorship care delivery.

Several limitations to our study need to be acknowledged. First, this cross-sectional study relies on survivor self-report instead of directly measuring PCP survivorship care delivery. Secondly, the use of an Internet-based survey creates selection bias in our study; cancer survivors who are over the age of 60, minorities, or those who have lower education are less likely to have Internet access and are expected to be under-sampled and under-represented in Internet-based research studies [35, 36]. Our research needs to be replicated in representative clinical or population based samples. Lastly, although our survey respondents have very heterogeneous tumor types and geographic representations, the smaller sample size in each of the specific categories prevents more in-depth comparison among subgroups.

## Conclusions

Despite its limitations, to our knowledge, this is the first Internet-based study of patient perspectives of cancer survivorship care delivery by primary care physicians. Through the use of a freely accessible Internet survey, our study allows for representation of cancer survivor populations that are otherwise under-represented in the current literature (e.g. in community setting, with non-breast cancer, rural, and other countries) on cancer survivorship care. The high trust between survivors and their PCPs builds a strong foundation towards optimal care. While cancer survivors in general are satisfied with care delivery by PCPs, they perceived that their PCPs have limited abilities in performing cancer-specific follow-up and late effect monitoring and treatment.Our findings highlight that equipping PCPs with cancer-specific knowledge, skills, and confidence is necessary to deliver high quality care for diverse groups of cancer survivors.

## Abbreviations

PCPs: Primary care physicians
PCP: Primary care physician
IOM: Institute of Medicine
PCDSCS: Primary care delivery of survivorship care scale
BA/BS: Bachelor of arts or science
